# Trends in pesticide suicide in South Korea, 1983–2014

**DOI:** 10.1017/S2045796019000118

**Published:** 2019-03-19

**Authors:** Eun Shil Cha, Shu-Sen Chang, Yeongchull Choi, Won Jin Lee

**Affiliations:** 1 Department of Preventive Medicine, Korea University College of Medicine, Seoul, South Korea; 2 Institute of Health Behaviors and Community Sciences, and College of Public Health, National Taiwan University, Taipei, Taiwan; 3 Seoul Workers’ Health Center, Seoul, South Korea

**Keywords:** Elderly, epidemiology, risk factors, suicide

## Abstract

**Aims:**

Self-poisoning using pesticides is among the major methods of suicide worldwide, and accounts for one-fifth of suicides in 2006–2010 in South Korea. We investigated long-term trends in pesticide suicide rates in South Korea and factors related to these trends.

**Methods:**

We calculated age-standardised rates of pesticide suicide in South Korea (1983–2014) using registered death data. We used graphical approach and joinpoint regression analysis to examine secular trends in pesticide suicide by sex, age and area, and a time-series analysis to investigate association of pesticide suicide rate with socioeconomic and agriculture-related factors. Age, period and cohort effects were examined using the intrinsic estimator method.

**Results:**

Age-standardised rate of pesticide suicide fluctuated between 1983 and 2000 before it markedly increased in 2000–2003 (annual percent change 29.7%), followed by a gradual fall (annual percent change −6.3%) in 2003–2011. Following the paraquat ban (2011–2012), there was a marked reduction (annual percent change −28.2%) in 2011–2014. Trend in pesticide suicide was associated with divorce rate but not with other factors studied. Declines in pesticide suicide in 2003–2011 were most noticeable in younger groups and metropolises; by contrast, elderly adults aged 70+ living in rural areas showed an upward trend until after the 2011–2012 paraquat ban, when it turned downward. In the age–period–cohort modelling, having been born between 1938 and 1947 was associated with higher pesticide suicide rates.

**Conclusions:**

Pesticide suicide trend changed substantially in South Korea over the last three decades. Effective prevention should include close monitoring of trends and strong regulations of toxic pesticides.

## Introduction

Globally, suicide is a significant public health problem. In 2012, an estimated 804 000 suicide deaths occurred worldwide, representing an annual global age-standardised suicide rate of 11.4 per 100 000 population (World Health Organization, 2014). Over the last three decades, South Korea has witnessed a dramatic increase in overall suicide rates – from 1983 to 2012, age-standardised suicide rates increased more than twofold in males (from 16.7 to 37.1 per 100 000) and threefold in females (from 5.6 to 17.7 per 100 000) (Lim *et al*., 2014). By 2012, South Korea's suicide rate was amongst the highest globally, with that among elderly individuals aged 70 years and above reaching 116 per 100 000 (World Health Organization, 2014).

Pesticide self-poisoning is a major contributor to the global burden of suicide (World Health Organization, 2014). Self-poisoning with pesticides was the causal method in approximately 30% and 14% of the world's suicides from 1990 to 2007 (Gunnell *et al*., 2007) and from 2010 to 2014 (Mew *et al*., 2017), respectively. South Korea has a relatively higher proportion of pesticide-related suicides as compared with other countries (Ajdacic-Gross *et al*., 2008). From 2006 to 2010, the average annual number of deaths from pesticide suicide was approximately 2700, accounting for 21% of all suicides in South Korea (Cha *et al*., 2014*a*). Among pesticides, paraquat was the most widely used herbicide (weed killer) (Cha *et al*., 2014*b*), had the highest fatality rate (78%) and was responsible for the largest proportion (35%) of poisoning cases in Korea (Rural Development Administration, 2008). The South Korean government cancelled the re-registration of paraquat from the end of November 2011 and has banned its sale since the end of October 2012 (http://www.rda.go.kr).

Although pesticide suicide is a major public health issue worldwide, only a few studies have investigated its long-term trends (Knipe *et al*., 2014) and factors that may influence the trends (Chang *et al*., 2012*a*); however, no study has yet analysed the independent effects of age, period and birth cohort using age–period–cohort modelling. Analysis of pesticide suicide trends may contribute to national suicide prevention strategies by identifying high-risk groups and potentially modifiable factors that influenced suicide trends. Accordingly, in this study, we investigated long-term trends in pesticide suicide rates in South Korea from 1983 to 2014 and factors related to these trends. We also aimed to distinguish the effects of age, time period and birth cohort on these trends.

## Methods

### Suicide and population data

Data on suicide rates and population counts in South Korea (1983–2014) were provided by Statistics Korea (http://kostat.go.kr). Our analyses used cause-of-death data from 1983 onwards, as this is the first year for which suicide data stratified by sex and age are available. These registered death data included information on age, sex, area of residence and date of death. The cause of death is coded using the *International Classification of Disease, Tenth Revision* (ICD-10); suicides by pesticide poisoning were identified using code X68. The year 1991 was the earliest year for which complete individual-level mortality data were available. Aggregate mortality data were available before 1991, but they were not stratified by area. Therefore, the analyses of area-specific trends in pesticide suicides were restricted to the period from 1991 to 2014. Because some pesticide suicides may have been misclassified as deaths due to other causes, including undetermined death by pesticide poisoning (Y18); accident by pesticide poisoning (X48); and suicide (X69), undetermined death (Y19) and accident (X49) by poisoning using unspecified chemicals (Cha *et al*., 2016*a*), we conducted a sensitivity analysis examining the impact of including these possible pesticide suicides on our findings. The five cause-of-death groups were selected based on findings from a previous study that showed the similarity in sociodemographic profiles between certified pesticide suicides and deaths in these alternative cause-of-death categories (Cha *et al*., 2016*a*).

### Data on influencing factors of pesticide suicide rates

Data on the sale of agricultural pesticides were extracted from the annual Agrochemical Year Books, published by the Korea Crop Protection Association (Korea Crop Protection Association, 2014). These data were based on reports from all companies that produce pesticides or import and synthesise related raw materials. Data on the proportion of the population living in households involved in farming were obtained from the agricultural census conducted every 5 years (http://kostat.go.kr). Estimated values for other years during the study period were based on linear interpolation. Information on paraquat regulations was included because paraquat is the most commonly involved pesticide in intentional pesticide poisoning. Information on paraquat regulations were obtained from the Korea Rural Development Administration (http://www.rda.go.kr). In South Korea, the re-registration of paraquat was cancelled from the end of November in 2011 and its sale was completely banned from the end of October in 2012. Information on potential risk factors for suicide such as unemployment rate (number of unemployed individuals per 100 labour force population), dependency ratio (ratio of the number of people aged 0–14 years plus the number aged 65 years and over to the number aged 15–64 years) and divorce rate (number of divorces per 1000 population) were collected from Statistics Korea. These socioeconomic factors have shown an association with overall suicide rates in South Korea (Ben Park and Lester, 2006; Chang *et al*., 2009).

### Statistical analysis

Age-standardised rates of pesticide suicide were calculated for each year based on the World Health Organization (WHO) world standard population (Ahmad *et al*., 2001). Age-specific rates were determined by analysing data for people aged 15–49, 50–59, 60–69 and 70+ years separately. These groups were chosen because pesticide suicide trends within these age bands have been reported to be generally similar (Cha *et al*., 2016*b*). Urbanisation levels were identified based on the administrative residential districts provided in the mortality data. Metropolises included the capital city (Seoul) and six other metropolitan cities. Small- and medium-sized cities and rural areas were determined by governmental administrative divisions, which are formed in consideration of population sizes and rural characteristics.

We used graphical approaches and joinpoint regression analysis to investigate trends in overall pesticide suicide rates and rates by sex, age and area. Joinpoint regression analysis allowed the identification of possible underlying trends comprising linear segments and ‘joinpoints’, (Kim *et al*., 2000) which, in this study, referred to the calendar years wherein any linear trend changed. The Joinpoint Regression Program (http://surveillance.cancer.gov/joinpoint) was used to estimate the annual percent change for linear trends and the location of joinpoints as well as their 95% confidence intervals (CIs).

We used time-series models to examine the associations between pesticide suicide rates and socioeconomic and agriculture-related factors. We fitted multivariable linear regression models using the Prais–Winsten estimation. This is a procedure meant to account for errors that are serially correlated and that follow a first-order autoregressive process (Prais and Winsten, 1954). The variables included were the amount of pesticide sold, farming household proportion, unemployment rate and divorce rate. The effects of the 1997 Asian economic crisis, the 2008 Great Recession and paraquat ban (2011–2012) were considered, as previous studies found that these factors influenced suicide trends in South Korea (Chang *et al*., 2009; Chan *et al*., 2014; Cha *et al*., 2016*b*), by including three dummy variables in the regression models. A value of 1 was assigned to each of the periods, namely, 1998–1999 (1997 Asian economic crisis), 2009–2010 (2008 Great Recession) and 2012–2014 (post-paraquat ban), and 0 for the remaining years during the study period. In this study, the periods 1998–1999 and 2009–2010 were considered the recessionary periods for the 1997 Asian economic crisis and the 2008 Great Recession, respectively, as previous studies indicated that the impact of both economic recessions were short-lived and were mainly seen within 1–2 years following the recessions in South Korea (Chang *et al*., 2009; Chan *et al*., 2014). Calendar year was included in the model to adjust for linear time trends. There may be time lags in the effects of the investigated factors on pesticide suicide rates. However, a certain level of impulsivity was observed in suicidal behaviours, particularly for pesticide self-poisoning, which frequently occurred soon after precipitating events (Conner *et al*., 2005). We thus focused on the same-year associations between the investigated factors and pesticide suicide rates. As pesticide suicide trends shifted from an upward trend to a downward one around 2003–2004 (Cha *et al*., 2016*a*), we also tested for differences in the association between the 1983–2003 and 2004–2014 periods by including interaction terms in the regression models.

An age–period–cohort model was used to analyse trends in pesticide suicide rates. An age–period–cohort analysis enables separation of the effects of age, period and cohort and helps to control for all three dimensions simultaneously (Yang *et al*., 2004). In other words, the age–period–cohort analysis is used to disentangle the relative contributions of the effects of chronological age, historical period and birth cohort on the rates of pesticide suicide in South Korea from 1983 to 2014. Although the size of each effect can be identified by calculating rates by age, cohort and period, or by joinpoint regression analysis, these methods are unable to separate the confounding effects of age, period and cohort induced by the exact linear dependency between the three factors. We used a novel method to fit the age–period–cohort model, namely, the intrinsic estimator (IE) method, which aims to deal with the non-identifiability problem (Yang *et al*., 2004). The dataset was first divided into fourteen 5-year age groups ranging from 15–19 years to 80+ years and six 5-year periods starting from 1983, except for one 7-year period (2008–2014). We then constructed consecutive 5-year birth cohorts, and data for each aggregate birth cohort were restructured according to the 14 age groups, each spanning 5 years. All analyses were performed using STATA (StataCorp, College Station, TX, USA). We fitted IE models to suicide mortality data using the *apc_ie* module in STATA.

## Results

### Pesticide suicide trends

The results of the joinpoint regression analysis showed that the overall pesticide suicide rate decreased slightly from 1983 to 1991 and increased somewhat from 1991 to 1995 (Table 1). It remained relatively stable from 1995 to 2000, and then rose markedly (annual percent change 29.7%) from 2000 to 2003, peaking at 9.2 per 100 000 in 2004. Pesticide suicide rates declined gradually from 2003 to 2011 (annual percent change −6.3%) and then decreased markedly from 2011 to 2014 (annual percent change −28.2%) following the paraquat ban in 2011–2012. The average annual number of pesticide suicide deaths was 1678 (ranging from 623 in 1991 to 3530 in 2004) among the population aged 15+ years (Supplementary Table 1S). In the sensitivity analysis, which included cause-of-death categories that might contain misclassified pesticide suicides from 1991 to 2014, we observed a continuous upward trend from 1991 to 2003, and the abrupt increase in pesticide suicide rates from 2000 was moderated (Supplementary Fig. 1S); by contrast, the change from a gradual decline in 2003–2011 to a marked reduction in 2011–2014 remained.

**Table 1.** Annual percent change (APC) and joinpoints (JP) and their 95% confidence intervals (95% CIs) from the joinpoint regression analysis of trends in pesticide suicide mortality rates in South Korea, 1983–2014

	Segment 1	JP1 (95% CI)	Segment 2	JP2 (95% CI)	Segment 3	JP3 (95% CI)	Segment 4	JP4 (95% CI)	Segment 5	JP5 (95% CI)	Segment 6
	APC (95% CI)		APC (95% CI)		APC (95% CI)		APC (95% CI)		APC (95% CI)		APC (95% CI)
Total	−5.3 (−8.9, −1.5)	1991 (1985, 1994)	14.4 (−2.8, 34.8)	1995 (1988, 2001)	0.8 (−7.1, 9.4)	2000 (1998, 2005)	29.7 (4.8, 60.4)	2003 (2002, 2009)	−6.3 (−8.5, −4.1)	2011 (2009, 2012)	−28.2 (−36.7, −18.6)
Sex											
Male	−6.9 (−10.6, −3.0)	1991 (1985, 1994)	15.9 (−2.3, 37.6)	1995 (1988, 2001)	1.2 (−7.0, 10.2)	2000 (1998, 2004)	30.3 (4.7, 62.1)	2003 (2002, 2009)	−5.6 (−7.8, −3.4)	2011 (2009, 2012)	−28.0 (−36.4, −18.4)
Female	3.2 (1.8, 4.6)	2000 (1998, 2001)	28.3 (−0.9, 66.1)	2003 (2002, 2005)	−7.1 (−9.7, −4.4)	2011 (2009, 2012)	−28.8 (−39.1, −16.7)				
Age group (male)											
15–49 years	−6.5 (−9.9, −3.0)	1991 (1985, 1994)	13.0 (−4.6, 34.0)	1995 (1988, 2002)	−2.1 (−12.1, 9.0)	2000 (1991, 2005)	21.8 (−13.3, 71.1)	2003 (2001, 2009)	−12.0 (−15.9, 8.0)	2011 (2008, 2012)	−35.1 (−45.2, −23.0)
50–59 years	−11.0 (−19.3, −1.8)	1989 (1987, 1993)	9.4 (6.7, 12.1)	2005 (2003, 2009)	−13.7 (−18.2, 9.0)						
60–69 years	10.9 (−4.5, 28.7)	1986 (1985, 1987)	−24.9 (−44.3, 1.2)	1989 (1988, 1991)	11.0 (8.5, 13.6)	2000 (1991, 2006)	22.8 (5.8, 42.6)	2004 (2002, 2009)	−7.4 (−12.0, −2.6)	2011 (2007, 2012)	−29.0 (−38.8, −17.6)
⩾70 years	15.8 (−2.2, 37.1)	1986 (1985, 1994)	−10.3 (−19.4, −0.2)	1991 (1988, 1998)	15.6 (11.4, 19.9)	2000 (1991, 2006)	35.3 (−3.5, 89.7)	2003 (2002, 2009)	0.5 (−4.0, 5.1)	2011 (2007, 2012)	−27.2 (−38.5, −13.8)
Age group (female)											
15–49 years	3.2 (1.8, 4.5)	2004 (2002, 2007)	−12.2 (−18.0, −6.0)	2012 (2009, 2012)	−48.0 (−68.8, −13.2)						
50–59 years	−5.4 (−11.7, 1.3)	1990 (1985, 2001)	9.9 (7.0, 12.9)	2004 (1998, 2007)	−5.7 (−13.6, 2.9)	2011 (2002, 2012)	−30.7 (−46.5, −10.2)				
60–69 years	4.3 (1.7, 7.0)	1997 (1985, 2003)	15.9 (8.0, 24.5)	2005 (2001, 2009)	−6.9 (−17.4, 4.8)	2011 (2007, 2012)	−33.3 (−48.8, −12.9)				
⩾70 years	−0.5 (−6.8, 6.1)	1992 (1989, 2000)	18.2 (13.9, 22.6)	2006 (2003, 2009)	−11.9 (−18.5, −4.8)						

**Fig. 1.**
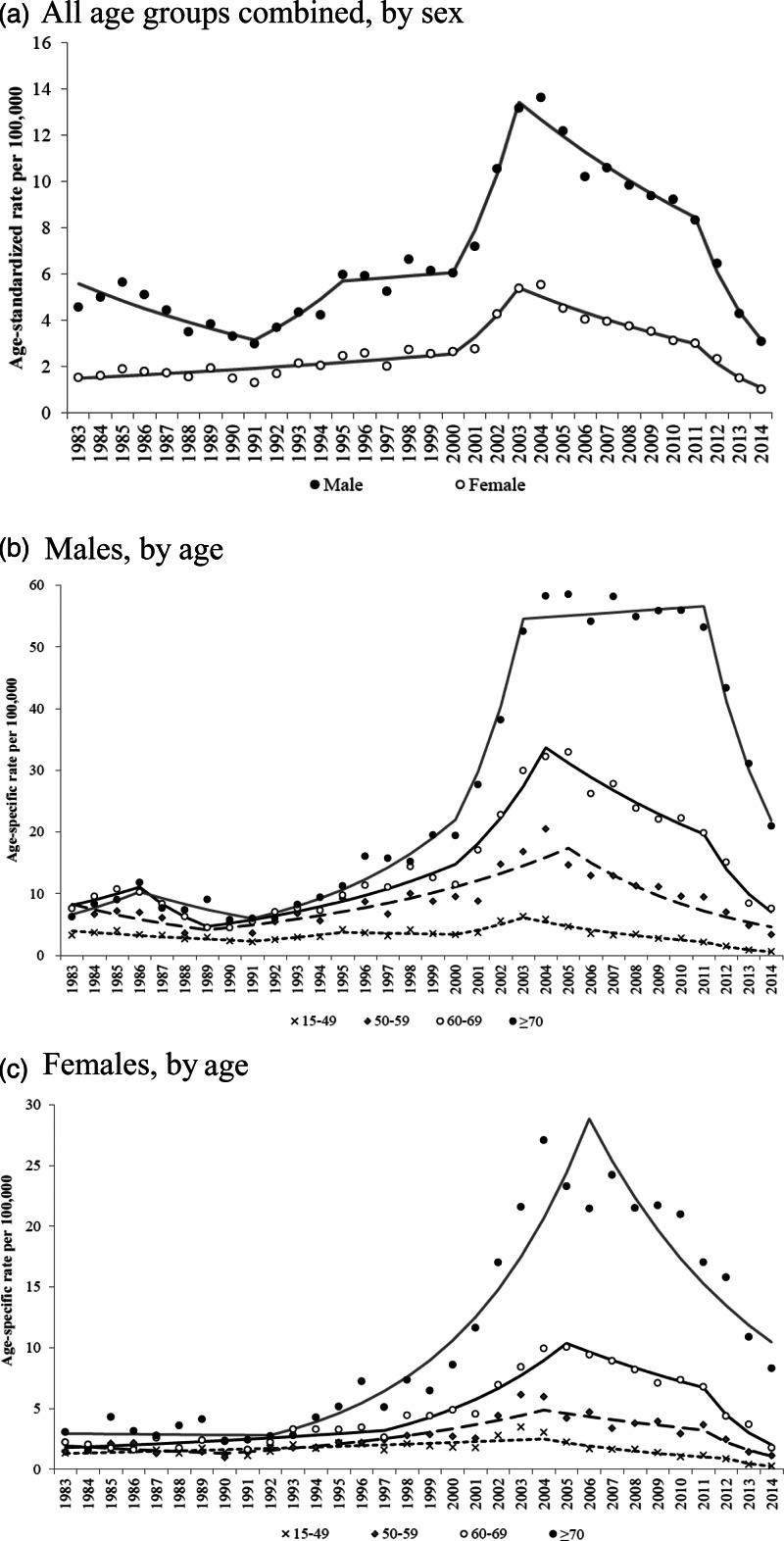
Trends in mortality rate of pesticide suicide by sex and age group in South Korea, 1983–2014. (*a*) All age groups combined, by sex. (*b*) Males, by age. (*c*) Females, by age. *Note*: Lines depict estimated linear trends from joinpoint regression analysis

Pesticide suicide rates were consistently higher in males than in females and generally increased with age (Fig. 1). Elderly males aged 70+ years living in rural areas had the highest rates of pesticide suicide during the study period, peaking at 112 per 100 000 in 2010 (Supplementary Fig. 2S). Before 2000, male pesticide suicide rates fluctuated somewhat while female rates showed an upward trend (Fig. 1*a*). By contrast, pesticide suicide trends were generally similar for both sexes after 2000. There were some variations in pesticide suicide trends across sex/age groups before 2000, while there were generally rapid increases in rates across sex/age groups between 2000 and 2003/2004 (Fig. 1*b* and 1*c*). Pesticide suicide rates declined in most sex/age groups after 2003/2004, although they remained high in males aged 70+. There was a marked reduction in pesticide suicide rates after 2011/2012 in all sex/age groups, and a joinpoint was found around 2011 or 2012 for all groups except males aged 50–59 and females aged 70+ (Table 1). The marked reduction in pesticide suicide after 2011 – when the re-registration of paraquat was cancelled – was most prominent in elderly males and females living in cities and rural areas. There was no change in trends around the same year in metropolises (Supplementary Fig. 2S).

### Factors that may influence the trends

**Fig. 2.**
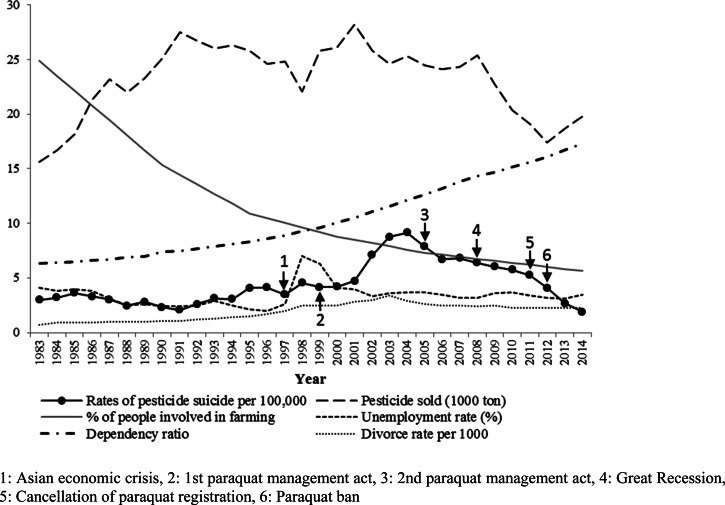
Trends in age-standardised rate of pesticide suicides and socioeconomic and agriculture-related factors in South Korea from 1983–2014.

Pesticide suicides rapidly decreased from 2011 compared with the downward trend from 2004 to 2010 (Fig. 2). The amount of pesticide sold increased from 1983 to 1991, fluctuated from 1991 to 2008, and declined after 2008. The proportion of the population living in farming households markedly declined over the study period. Unemployment rates peaked in 1998 (7.0%) following the 1997 Asian economic crisis and fluctuated (3.1%–4.1%) from 2000 to 2014. The dependency ratio steadily increased during the study period. Divorce rates increased markedly from 1983 to 2003 and gradually decreased thereafter. Pesticide suicide rate was positively associated with farming household proportion and divorce rate in the models controlling for calendar year; however, after adjusting for all covariates, only divorce rate remained significant (Table 2). The associations between farming population or dependency ratio and pesticide suicide differed between the periods 1983–2003 and 2004–2014. However, the statistical evidence for the interaction effect of period in the associations was substantially lessened after fully controlling for all factors investigated in Model 2. Therefore, there was limited evidence that the associations differed between the two time periods.

**Table 2.** Coefficients (95% confidence intervals) from Prais–Winsten regression analyses of the associations between socioeconomic and agriculture-related factors and the mortality rates of pesticide suicide in South Korea, 1983–2014

Factors	Pesticide suicide			
	Model 1^a^	*p* for interaction with period^b^	Model 2^c^	*p* for interaction with period^b^
Amount of pesticide sold	−0.11 (−0.29, 0.07)	0.102	−0.15 (−0.37, 0.06)	0.727
% population living in households involved in farming	1.61 (0.23, 2.99)	<0.001	−0.21 (−1.43, 1.01)	0.081
Unemployment rate	0.16 (−0.15, 0.48)	0.828	−0.15 (−0.91, 0.61)	0.787
Dependency ratio	2.53 (−0.05, 5.12)	<0.001	0.04 (−3.20, 3.78)	0.056
Divorce rate	2.19 (0.75, 3.62)	0.489	2.03 (0.28, 3.78)	0.468
Asian economic crisis	0.50 (−0.67, 1.68)	–	0.25 (−2.44, 2.94)	–
Great Recession	0.04 (−1.16, 1.23)	–	−0.06 (−1.28, 1.17)	–
Paraquat ban	−1.32 (−3.03, 0.40)	–	−1.47 (−3.22, 0.29)	–

a Adjusted for calendar year only.

b *p*-value for a difference in the association between the periods of 1983–2003 and 2004–2014.

c Adjusted for calendar year and all covariates in table.

### Age, period and cohort effects

**Fig. 3.**
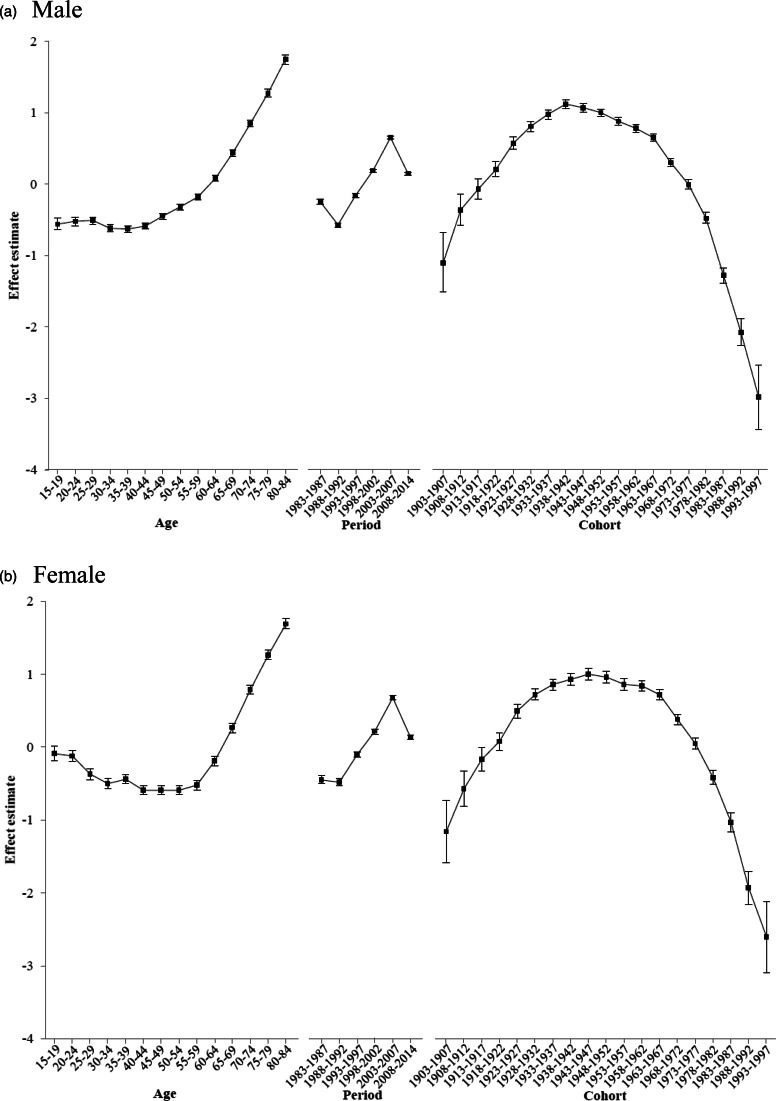
Age, period and birth cohort effects of mortality rate of pesticide suicide by sex in South Korea, 1983–2014. (*a*) Male. (*b*) Female. *Note*: Because the intrinsic estimator does not use reference categories for the age, period and cohort coefficients, the *y*-axis for each effect represents the natural logarithm of the relative mortality rate of suicide at the age, calendar year of death or birth year of the deceased. For example, the value of 1.12 for the 1938–1942 birth cohort of males indicates that membership in this cohort nearly tripled risk (e^1.12^  = 3.06) of pesticide suicide compared with that of all cohorts combined, and the effect is independent of period and age effects.

The contributions of age, period and cohort effects on observed pesticide suicide trends, controlling simultaneously for all three parameters, are shown in Fig. 3. Age was an important factor contributing to trends in pesticide suicide in South Korea. The age effects increased steadily from 60 years upward in both sexes, peaking for the oldest age group, after adjusting for the cohort and period effects. The risk in the group aged 80–84 was about six (*β*_male_  = 1.75, e^1.75^ = 5.75) and five times (*β*_female_  = 1.69, e^1.69^ = 5.42) higher in males and females, respectively, than that for all ages combined. After a decline in pesticide suicide mortality from 1983 to 1987, period effects increased over the next two decades, peaking between 2003 and 2007 (*β*_male_  = 0.65, e^0.65^ = 1.92; *β*_female_  = 0.68, e^0.68^ = 1.97), which was then followed by a marked fall from 2008 to 2014 in both sexes. The cohort effects were highest between the 1920s and 1960s, peaking in the cohorts born between 1938 and 1942 for males (*β*_male_  = 1.12, e^1.12^ = 3.06) and between 1943 and 1947 for females (*β*_female_  = 1.00, e^1.00^ = 2.72).

## Discussion

Pesticide suicide rates changed markedly over the last 30 years in South Korea. The age-standardised pesticide suicide mortality rate fluctuated between 1983 and 2000 before markedly increasing from 2000 to 2003, peaking at 9.2 per 100 000 in 2004; it gradually decreased afterwards and rapidly declined following the 2011–2012 ban of paraquat. Pesticide suicide rates were higher in males than they were in females, increased with age, and were higher in rural than they were in non-rural areas. The increase in pesticide suicides before the mid-2000s was greatest in elderly adults aged 70+ years. Pesticide suicide rates decreased in most sex/age groups across all areas after the mid-2000s, but they continued increasing among elderly adults living in rural areas, among whom the 2011–2012 paraquat ban also had the largest impact on pesticide suicide trends. From the age–period–cohort analysis, there was a peak in the generation born between 1938 and 1947 and the relative risk was nearly three times the risk of all birth cohorts combined, independent of period and age effects.

The increase in pesticide suicides in the 2000s in South Korea is unique compared with other industrialised Asian countries such as Japan (Kim *et al*., 2011) and Taiwan (Chang *et al*., 2012*a*), or countries in other regions (Ajdacic-Gross *et al*., 2008). In Japan, pesticide ingestion accounted for only 1.6–3.8% of all suicides in 2006 (Kim *et al*., 2011). In Taiwan, there was a marked reduction (67%) in pesticide suicides from 1987 to 2010, attributable to a decline in the farming population of a similar magnitude (Chang *et al*., 2012a). The high pesticide suicide mortality rates until the mid-2000s in South Korea might have been due to the availability of highly lethal pesticides (Seok *et al*., 2009). The highest pesticide suicide rates in rural areas could be largely explained by accessibility to pesticides. A similar phenomenon was also found in other Asian countries such as Taiwan (Chang *et al*., 2012*b*), China (Kong and Zhang, 2010) and Sri Lanka (Eddleston *et al*., 2006). Although paraquat began to be somewhat regulated in 1999, which involved setting standards for handling, compulsory education in pesticide use for farmers and sellers and enforcement of labelling, persistently high pesticide suicide rates in the 2000s indicates that these regulations had little effect on reducing pesticide suicides. The lack of strict legislative regulations to reduce access to high toxicity pesticides indeed might be responsible for the high mortality rate for pesticide poisonings in South Korea (Lee *et al*., 2009; Rural Development Administration, 2013; Moon *et al*., 2016). Furthermore, cultural and environmental factors could also contribute to the choice of suicide methods (Wu *et al*., 2012).

The rise in pesticide suicides before the mid-2000s was most prominent among elderly adults. South Korea's increasing suicide rates since the 1990s was previously found to be most prominent in older people (Kim *et al*., 2010; Hong and Knapp, 2014), and pesticide ingestion was the most common suicide method among elderly individuals in South Korea (Lee *et al*., 2009; Kim *et al*., 2015). This makes it a critical factor contributing to the marked rise in elderly suicide in the country. Other contributing factors include the unpreparedness of the Korean society for a rapidly ageing population (Kim *et al*., 2011). Such rapid population ageing has made it difficult to develop proper social welfare systems for the elderly. Insufficient social safety networks and relative poverty might play important roles in making the elderly population more vulnerable to suicide risk in South Korea (Kim *et al*., 2011). In fact, more than 45% of people aged 65 or over were in relative poverty (i.e. having incomes below one-half of the median household income) in South Korea in 2005. This was the highest poverty rate among OECD countries (OECD, 2011). Weakening family support and social integration during rapid industrialisation, including the breakdown of the traditional family structure, may be additional contributors to the rise in elderly suicide in South Korea (Kim *et al*., 2011). In South Korea, indicators of social integration such as the divorce rate were also found to be high in the elderly population (Ben Park and Lester, 2006; Hong and Knapp, 2014), which accords with our findings that a change in divorce rate was most consistently associated with a change in pesticide suicide rates.

The finding that the abrupt increase in pesticide suicide rates from around 2000 was moderated in the sensitivity analysis, which included data on pesticide suicide cases that were possibly misclassified, indicates that an improvement in the quality and comprehensiveness of suicide data around 2000 might have contributed to the increase in pesticide suicide rates. Certified pesticide suicides markedly increased from 2000 to 2003 in South Korea, which was a continuation of a previous upward trend from 1991 when we included possible misclassified pesticide suicides (Cha *et al*., 2016*a*). Because the accuracy of cause-of-death data has improved since 2000 in South Korea due to the linkage of registered death data to a number of administrative datasets (Statistics Korea, 2016), the improved data reliability might have partially contributed to the increase in pesticide suicide mortality from 2000 to 2003. A recent study using South Korean data also showed that 43% of the increase in overall suicide mortality from 1992 to 2011 was related to a decrease in accidental deaths (Chan *et al*., 2015). A Taiwanese study also indicated that changes in data quality or death certification practices might influence trends in pesticide suicide (Chang *et al*., 2010).

A decrease in pesticide suicide rates was observed in South Korea from 2004 to 2011, particularly among people living in metropolises and cities and in those aged below 70 years. However, this decrease was accompanied by a rise in use of hanging, particularly in females aged 20–59 years (Jee and Jo, 2014), and gas poisoning, particularly in the younger population (Hee Ahn *et al*., 2012; Choi *et al*., 2014). A decrease in the popularity of pesticide ingestion and a shift towards other suicide methods, particularly among the younger population, might have contributed to the decrease in pesticide suicide since the mid-2000s in South Korea. Our data somewhat supported this, showing a continuous rise in non-pesticide suicide rates as pesticide suicide rates declined from 2003/2004 (Supplementary Fig. 1S). A swath of celebrity suicides by hanging and gas poisoning following the mid-2000s in South Korea might have also influenced the choice of suicide method, particularly in young people (Fu and Chan, 2013; Ji *et al*., 2014). Due to less accessibility to pesticides in urban areas, the shift from pesticide ingestion to other suicide methods may be more pronounced in urban areas compared with rural ones. In addition, because young people tend to live in urban areas, urban residents may be more readily influenced by mass media reporting of new trends in methods of suicide, such as carbon monoxide poisoning. Previous studies indicated a smaller risk of carbon monoxide suicide in rural areas compared with metropolitan areas in the period after the epidemic of suicides by carbon monoxide poisoning (Choi *et al*., 2014).

The finding that the paraquat ban influenced pesticide suicide trends was consistent with findings from previous analyses using data up to 2013 (Cha *et al*., 2016*b*; Myung *et al*., 2015). Our data showed that the downward trend continued in 2014. We also showed a marginally significant negative association between pesticide suicide and the paraquat ban (coefficient  = −1.47, 95% CI −3.22 to 0.29). A previous study similarly showed that South Korea's pesticide suicide rates showed a further dip in an already downward trend following the paraquat ban (Cha *et al*., 2016*b*). Our sex-/age-/area-specific analyses showed that this was evident in most groups except elderly individuals living in rural areas, where there was a reverse from an upward to a downward trend around the time paraquat was banned. The high rural elderly suicide rates in South Korea might have continued to rise if paraquat had not been banned, suggesting that the ban had a substantial impact on this group. Internationally, it is also widely recognised that regulatory action to ban highly hazardous pesticides, particularly organophosphate insecticides and paraquat, was followed by falls in pesticide suicides and overall suicides (Gunnell *et al*., 2017). The present study also indicated that there was no increase in non-pesticide suicide after the paraquat ban (Supplementary Fig. 1S), providing further evidence for the effectiveness of restricting the means for suicide in order to prevent it and that there is no evidence for significant substitution of methods. If suicidal acts around crisis over a short duration could be effectively avoided through restricting access to a lethal pesticide, suicidal attempts using other means may not occur because the suicidal urge may pass quickly (Mishara, 2007). Even when individuals who attempted suicide by pesticide ingestion shifted to using other, less lethal pesticides (Lee *et al*., 2015; Moon *et al*., 2016), their likelihood of survival increased substantially when paraquat was unavailable (Moon *et al*., 2016). However, future research into trends in fatal and non-fatal pesticide self-poisoning, as well as longer-term trends in method-specific suicide rates, is needed.

Males are generally more vulnerable to suicide risk than females and male suicide rates were more sensitive to socioeconomic factors than female rates (Girard, 1993). The fluctuations in pesticide suicide trends among males before 2000 observed in the present study could be explained by rapid socioeconomic changes in South Korea in the 1980s and 1990s. For example, a previous study indicated that the increase in male suicide, particularly in the middle-aged and older group, in South Korea in 1983–1986 may be related to the pressure resulted from the competitive, achievement-oriented working environments during a period of rapid economic development (Roh, 2013). By contrast, another previous study comparing the pattern of suicide in South Korea and Japan suggested that female suicide rates were less affected by the economic cycle than male rates (Kim *et al*., 2011).

The results from the age–period–cohort analysis were somewhat different from those of previous studies on overall suicides in South Korea (Lee, 2008; Jeon *et al*., 2016; Park *et al*., 2016). The period effects indicating an abrupt decrease in pesticide suicide mortality from 2008 to 2014 contrast sharply with the steadily increasing trend in overall suicide mortality (Jeon *et al*., 2016; Park *et al*., 2016). Part of the decrease in pesticide suicide between 2008 and 2014 was contributed by the marked reduction in 2012–2014 following the paraquat ban in 2011–2012 (Myung *et al*., 2015; Cha *et al*., 2016*b*). We also found a higher pesticide suicide risk among the birth cohorts born between the 1920s and 1960s, which is roughly similar to findings of previous studies on overall suicides in South Korea (Lee, 2008; Jeon *et al*., 2016; Park *et al*., 2016). Cohort effects for pesticide suicide mortality were highest for those born between 1938 and 1947; this cohort has life experiences such as the increasing availability of toxic pesticides during their young adulthood that may predispose them to choosing pesticides as a suicide method. In South Korea, pesticides began to be used extensively in the late 1950s (Korea Crop Protection Association, 1993) during the young adulthood of cohorts born in the 1940s. The cohort effect was similarly found in a Sri Lankan study (Knipe *et al*., 2014).

We have conducted in-depth analyses such as joinpoint regression analysis, Prais–Winsten regression and age–period–cohort analysis to identify underlying trends and the factors that may have affected the trends in pesticide suicides in South Korea. The limitations of the study include the ecological design, the linear trend assumption of joinpoint and Prais–Winsten regression analyses, and the unavailability of data pertaining to other potential risk factors for suicide.

In conclusion, pesticide suicide trends changed substantially in South Korea over the last three decades and varied across demographic groups. In particular, the ban on paraquat appeared to be the most effective approach to reducing suicide in the male elderly population, who were at high risk of pesticide suicide. Therefore, strong regulations on toxic pesticides may help ameliorate the very high rates of suicide mortality observed in this population.

## Supplementary material

For supplementary material accompanying this paper visit http://dx.doi.org/10.1017/S2045796019000118. click here to view supplementary material

## Availability of data and materials

All data used in this manuscript are publicly available. Data on suicide rates and population counts in South Korea (1983–2014) were provided by Statistics Korea (http://kostat.go.kr).
