# Supplementary material for: Trends in pesticide suicide in South Korea, 1983–2014
Source: Epidemiol Psychiatr Sci. 2019 Mar 19;29:e25. doi: 10.1017/S2045796019000118 (PMC8063219; doi:10.1017/S2045796019000118)
Supplement: Supplementary file 1 [file S2045796019000118sup001.docx]

**Supplementary materials**

**Table 1S**. The number of pesticide suicides and population aged 15 or older in South Korea (1983–2014)

|  | 1983 | | 1984 | | 1985 | | 1986 | | 1987 | | 1988 | | 1989 | | 1990 | |
| --- | --- | --- | --- | --- | --- | --- | --- | --- | --- | --- | --- | --- | --- | --- | --- | --- |
|  | Pesticide suicides | Population count | Pesticide suicides | Population count | Pesticide suicides | Population count | Pesticide suicides | Population count | Pesticide suicides | Population count | Pesticide suicides | Population count | Pesticide suicides | Population count | Pesticide suicides | Population count |
| Total | 742 | 27109435 | 830 | 27814196 | 950 | 28501202 | 878 | 29183841 | 840 | 29875313 | 699 | 30543802 | 813 | 31187907 | 698 | 32256057 |
| Sex |  |  |  |  |  |  |  |  |  |  |  |  |  |  |  |  |
| Male | 541 | 13510834 | 610 | 13863956 | 686 | 14217259 | 625 | 14546242 | 589 | 14879849 | 470 | 15202258 | 521 | 15512462 | 463 | 15990514 |
| Female | 201 | 13598601 | 220 | 13950240 | 264 | 14283943 | 253 | 14637599 | 251 | 14995464 | 229 | 15341544 | 292 | 15675445 | 235 | 16265543 |
| Age |  |  |  |  |  |  |  |  |  |  |  |  |  |  |  |  |
| 15–49 | 511 | 21859606 | 589 | 22347352 | 659 | 22803710 | 582 | 23269439 | 583 | 23720333 | 486 | 24133248 | 576 | 24511537 | 474 | 25303888 |
| 50–59 | 118 | 2699521 | 114 | 2815628 | 132 | 2945338 | 136 | 3078498 | 115 | 3222598 | 87 | 3369072 | 103 | 3511584 | 113 | 3632871 |
| 60–69 | 74 | 1610037 | 89 | 1668340 | 98 | 1722513 | 95 | 1774538 | 93 | 1830316 | 70 | 1896280 | 65 | 1977854 | 67 | 2057373 |
| ≥ 70 | 39 | 940271 | 38 | 982876 | 61 | 1029641 | 65 | 1061366 | 49 | 1102066 | 56 | 1145202 | 69 | 1186932 | 44 | 1261925 |
|  | 1991 | | 1992 | | 1993 | | 1994 | | 1995 | | 1996 | | 1997 | | 1998 | |
|  | Pesticide suicides | Population count | Pesticide suicides | Population count | Pesticide suicides | Population count | Pesticide suicides | Population count | Pesticide suicides | Population count | Pesticide suicides | Population count | Pesticide suicides | Population count | Pesticide suicides | Population count |
| Total | 623 | 32256057 | 820 | 33750335 | 1008 | 34255716 | 1001 | 34802329 | 1367 | 35380553 | 1367 | 35953819 | 1174 | 36526385 | 1576 | 36751666 |
| Sex |  |  |  |  |  |  |  |  |  |  |  |  |  |  |  |  |
| Male | 419 | 15990514 | 539 | 16740905 | 643 | 16993595 | 651 | 17255311 | 933 | 17540739 | 910 | 17819417 | 809 | 18098414 | 1072 | 18207569 |
| Female | 204 | 16265543 | 281 | 17009430 | 365 | 17262121 | 350 | 17547018 | 434 | 17839814 | 457 | 18134402 | 365 | 18427971 | 504 | 18544097 |
| Age |  |  |  |  |  |  |  |  |  |  |  |  |  |  |  |  |
| 15–49 | 420 | 25303888 | 529 | 26195173 | 654 | 26456199 | 640 | 26788391 | 871 | 27182385 | 794 | 27535198 | 657 | 27790459 | 878 | 27865754 |
| 50–59 | 90 | 3632871 | 144 | 3943076 | 169 | 4036004 | 149 | 4078818 | 228 | 4070567 | 220 | 4096086 | 190 | 4214981 | 269 | 4259274 |
| 60–69 | 67 | 2057373 | 91 | 2162333 | 114 | 2237984 | 117 | 2360985 | 150 | 2498579 | 180 | 2637600 | 173 | 2763198 | 248 | 2827482 |
| ≥ 70 | 46 | 1261925 | 56 | 1449753 | 71 | 1525529 | 95 | 1574135 | 118 | 1629022 | 173 | 1684935 | 154 | 1757747 | 181 | 1799156 |

(Continued)

|  | 1999 | | 2000 | | 2001 | | 2002 | | 2003 | | 2004 | | 2005 | | 2006 | |
| --- | --- | --- | --- | --- | --- | --- | --- | --- | --- | --- | --- | --- | --- | --- | --- | --- |
|  | Pesticide suicides | Population count | Pesticide suicides | Population count | Pesticide suicides | Population count | Pesticide suicides | Population count | Pesticide suicides | Population count | Pesticide suicides | Population count | Pesticide suicides | Population count | Pesticide suicides | Population count |
| Total | 1456 | 37170729 | 1483 | 37569140 | 1705 | 37952596 | 2627 | 38298236 | 3299 | 38613892 | 3530 | 38940565 | 3124 | 39321365 | 2747 | 39758636 |
| Sex |  |  |  |  |  |  |  |  |  |  |  |  |  |  |  |  |
| Male | 979 | 18411678 | 980 | 18608047 | 1166 | 18798753 | 1773 | 18968672 | 2197 | 19123726 | 2356 | 19288034 | 2130 | 19484318 | 1831 | 19711677 |
| Female | 477 | 18759051 | 503 | 18961093 | 539 | 19153843 | 854 | 19329564 | 1102 | 19490166 | 1174 | 19652531 | 994 | 19837047 | 916 | 20046959 |
| Age |  |  |  |  |  |  |  |  |  |  |  |  |  |  |  |  |
| 15–49 | 763 | 27988068 | 732 | 28097158 | 773 | 28212733 | 1191 | 28230324 | 1389 | 28174868 | 1254 | 28120647 | 976 | 28019150 | 733 | 27933357 |
| 50–59 | 250 | 4335489 | 267 | 4407216 | 251 | 4450388 | 430 | 4499575 | 528 | 4609405 | 632 | 4785260 | 479 | 5079574 | 477 | 5411368 |
| 60–69 | 237 | 2964704 | 242 | 3097799 | 329 | 3236454 | 483 | 3416878 | 650 | 3565446 | 734 | 3640881 | 757 | 3674785 | 637 | 3701995 |
| ≥ 70 | 206 | 1882468 | 242 | 1966967 | 352 | 2053021 | 523 | 2151459 | 732 | 2264173 | 910 | 2393777 | 912 | 2547856 | 900 | 2711916 |
|  | 2007 | | 2008 | | 2009 | | 2010 | | 2011 | | 2012 | | 2013 | | 2014 | |
|  | Pesticide suicides | Population count | Pesticide suicides | Population count | Pesticide suicides | Population count | Pesticide suicides | Population count | Pesticide suicides | Population count | Pesticide suicides | Population count | Pesticide suicides | Population count | Pesticide suicides | Population count |
| Total | 2880 | 40248599 | 2799 | 40762825 | 2741 | 41272778 | 2719 | 41750637 | 2580 | 42205994 | 2103 | 42636198 | 1442 | 43037651 | 1072 | 43426251 |
| Sex |  |  |  |  |  |  |  |  |  |  |  |  |  |  |  |  |
| Male | 1937 | 19965899 | 1879 | 20235429 | 1838 | 20501764 | 1875 | 20746013 | 1764 | 20975553 | 1423 | 21188643 | 978 | 21385918 | 736 | 21578663 |
| Female | 943 | 20282700 | 920 | 20527396 | 903 | 20771014 | 844 | 21004624 | 816 | 21230441 | 680 | 21447555 | 464 | 21651733 | 336 | 21847588 |
| Age |  |  |  |  |  |  |  |  |  |  |  |  |  |  |  |  |
| 15–49 | 692 | 27879053 | 709 | 27807152 | 579 | 27692755 | 534 | 27487268 | 451 | 27239550 | 322 | 26998474 | 169 | 26797618 | 118 | 26607569 |
| 50–59 | 464 | 5701710 | 451 | 5991652 | 477 | 6324326 | 421 | 6726716 | 471 | 7175714 | 357 | 7537309 | 245 | 7786325 | 179 | 8000584 |
| 60–69 | 672 | 3789400 | 608 | 3909751 | 568 | 4006145 | 591 | 4092726 | 541 | 4150539 | 402 | 4217736 | 260 | 4343385 | 208 | 4529947 |
| ≥ 70 | 1052 | 2878436 | 1031 | 3054270 | 1117 | 3249552 | 1173 | 3443927 | 1117 | 3640191 | 1022 | 3882679 | 768 | 4110323 | 567 | 4288151 |


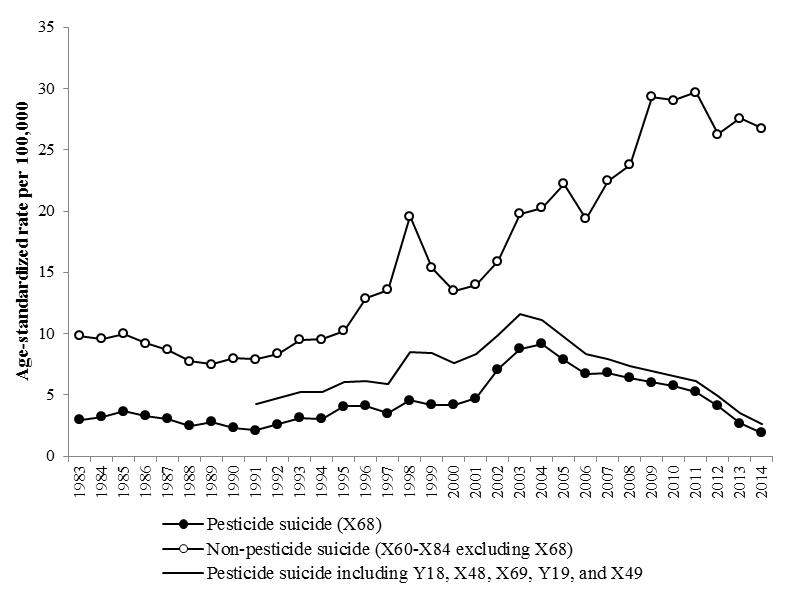


**Figure 1S**. Trends in age-standardized mortality rate of suicides in South Korea, 1983–2014

**Figure 2S.** Trends in mortality rate of pesticide suicide by sex, age group, and area in South Korea, 1991–2014

(a) Male, by age


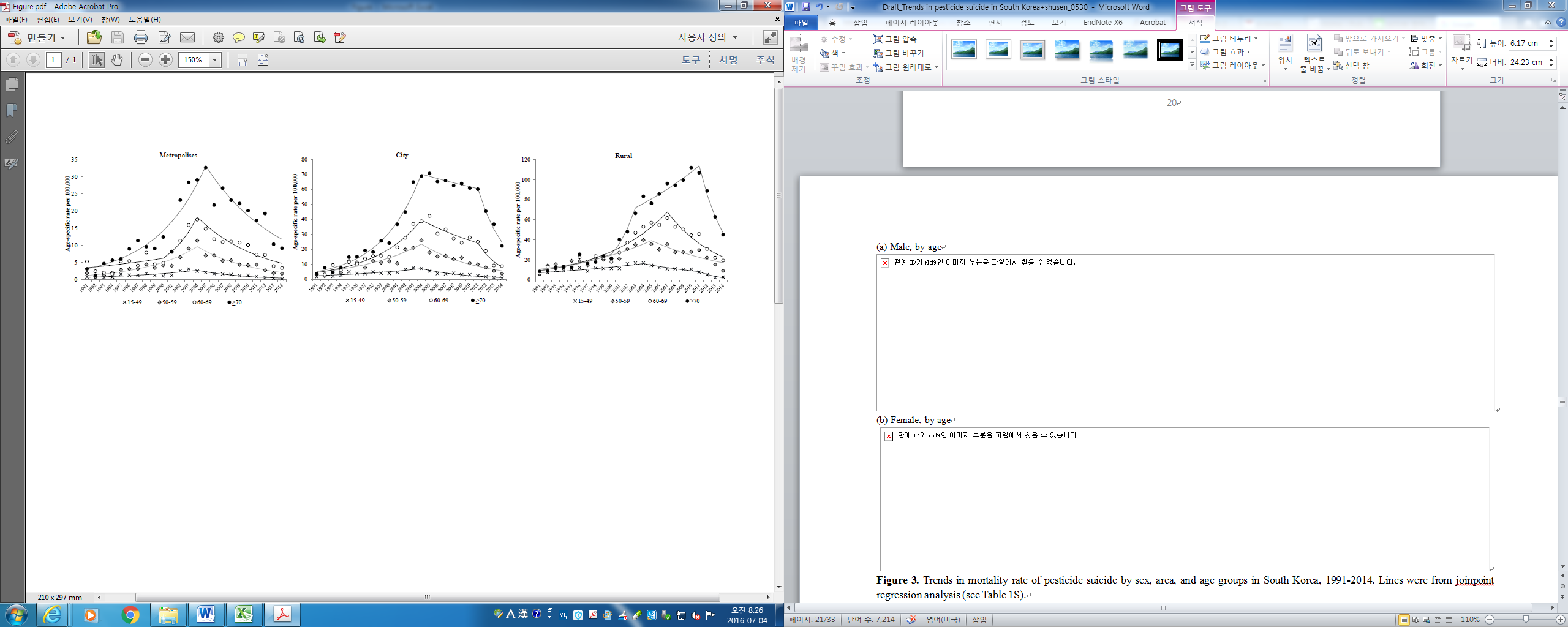


(b) Female, by age


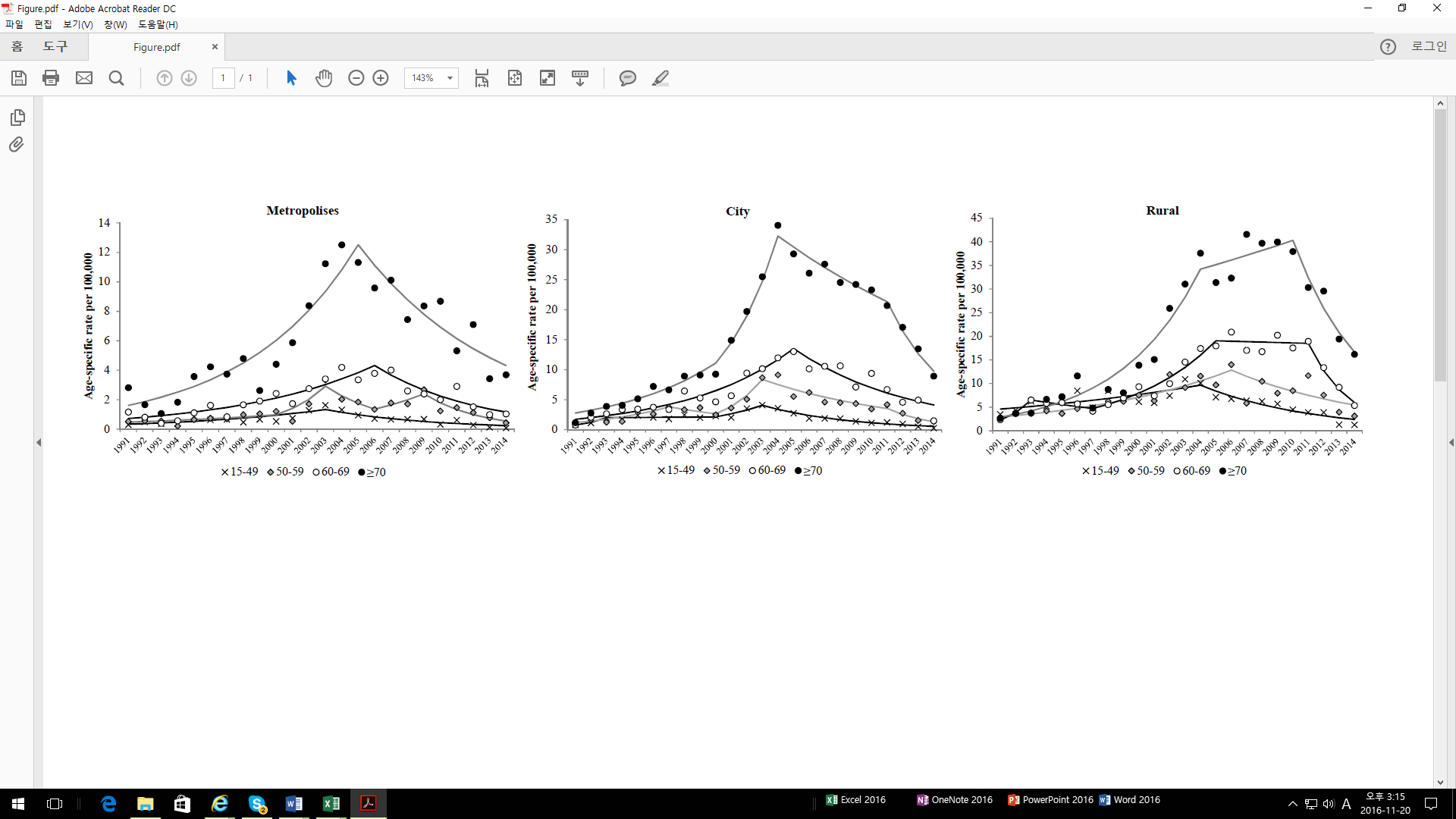


Note: Using the administrative residential districts provided in the mortality data, we identified three urbanization levels: (i) “metropolises,” including Seoul and six major cities (ii) “small- and medium-sized cities” and (iii) “rural areas.” Cities and rural areas were determined by considering population sizes, with cities requiring a population of more than 50,000. Lines depict estimated linear trends from joinpoint regression analysis.
